# Multifunctional Roles of Autophagy in Fungi

**DOI:** 10.3390/jof12050377

**Published:** 2026-05-20

**Authors:** Aron Osakina, William J. Steinbach, Praveen R. Juvvadi

**Affiliations:** Division of Pediatric Infectious Diseases, Department of Pediatrics, Arkansas Children’s Research Institute, University of Arkansas for Medical Sciences, Little Rock, AR 72202, USA; aosakina@uams.edu (A.O.); wsteinbach@uams.edu (W.J.S.)

**Keywords:** fungi, autophagy, ATGs, autophagosome, mitophagy, golgiphagy, pexophagy, antifungals, TOR, drug resistance

## Abstract

Autophagy, also referred to as the “self-eating machinery”, is a crucial process where organisms maintain intracellular homeostasis through recycling or degrading non-essential and damaged cellular components. It is important in numerous biological functions such as cellular differentiation, aging, nutrient sensing, stress response, tissue homeostasis, immunity, and programmed cell death. Autophagy induction occurs with the formation of a double-layered membrane structure called “autophagosome”. The autophagosome wraps damaged organelles or proteins and transports them to the vacuole or lysosome for degradation. Autophagy is beneficial to organisms, and it should be optimally regulated because elevated or decreased levels are detrimental for survival. To date, more than 40 autophagy-related genes (ATGs) have been identified in the budding yeast *Saccharomyces cerevisiae*, with most having homologs in fungi and higher eukaryotes. Majority of the ATGs in industrial and pathogenic fungal species have been characterized and known to play vital roles in growth, development, and virulence. In this review we provide a comprehensive overview of ATGs in various fungal species and highlight how autophagy is regulated and controls various functions in plant, human, and industrial fungal species.

## 1. Introduction

Autophagy is an evolutionarily conserved process where cytosolic material, including organelles and autophagy-related proteins, is sequestered by structures called “autophagosomes” and subsequently delivered to vacuoles as autophagic bodies [1]. Autophagy may be broadly classified into non-selective and selective autophagy based on the type of cellular material that is directed for degradation. Non-selective autophagy, also called the cargo-independent process, is induced under starvation conditions to recycle bulk cytosolic materials [2]. On the other hand, selective autophagy, also referred to as cargo-dependent autophagy, is independent of starvation to selectively degrade and recycle damaged organelles or proteins and maintain intracellular homoeostasis [3]. While non-selective and selective autophagy processes utilize the same mechanism to form autophagosomes, the latter relies on specific autophagic factors that recognize cargos and membrane components.

**Figure 1:**
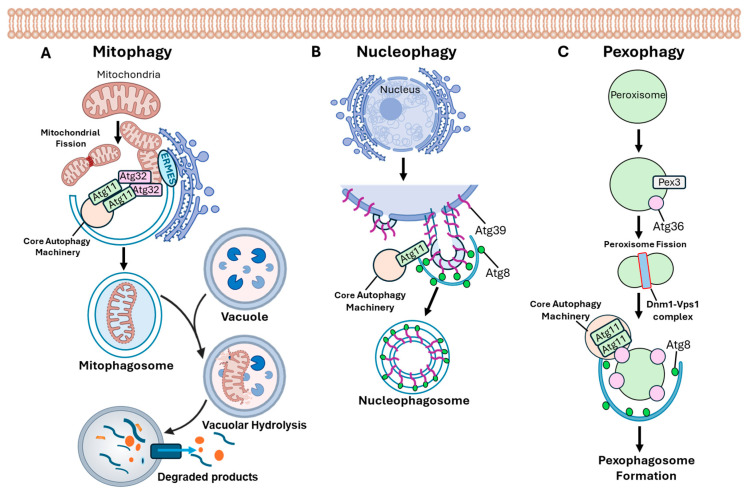
**Selective autophagy mechanisms showing autophagic degradation of mitochondria, nuclei, and peroxisomes.** (**A**) Following mitochondrial fission, the mitophagy transmembrane receptor Atg32 localized on the mitochondrial outer membrane interacts with Atg11. Atg11 tethers the mitochondria to the PAS for selective sequestration of mitochondria. The endoplasmic reticulum–mitochondria encounter structure (ERMES) complex acts as a tether between the ER and mitochondria. Extension of the isolation membrane leads to mitophagosome formation and fusion with the vacuole for degradation by vacuolar hydrolases. The degraded products are released for recycling of cellular material. (**B**) During nucleophagy, Atg39 receptor interacts with Atg8 through an Atg8 interacting motif in its N-terminal cytosolic tail, and upon nucleophagy induction, it binds to Atg8 puncta to form the nucleophagosome destined for degradation of nuclear material. (**C**) In the pexophagy process, the peroxisomal membrane protein Pex3 acts as a peroxisomal ligand and recruits the Atg36 receptor protein to the peroxisomal membrane. Atg36 binds to Atg11 and recruits peroxisomal fission complexes containing the dynamin-related GTPases Dnm1 and Vps1 to target peroxisomes, thereby facilitating their sequestration by phagophores. Following pexophagosome formation, fusion with the vacuolar membrane releases peroxisomes into the vacuolar lumen for degradation. Note: Panels illustrated do not show complete stages in the autophagic degradation process. Individual components in the “core autophagy machinery” are not shown. “Core autophagy machinery” comprises the initial ATG1-ATG13-ATG17 complex including Atg1, Atg11, Atg13, Atg17, Atg29 and Atg31 proteins which regulate the induction of autophagosome formation, the Atg9 and its cycling system including Atg2, Atg9 and Atg18 proteins that function in membrane delivery to the expanding phagophore, the PtdIns 3-kinase (PtdIns3K) complex including Vps34, Vps15, Vps30/Atg6, and Atg14 proteins that function in vesicle nucleation, and the ubiquitin-like conjugation systems including the Atg12 (Atg5, Atg7, Atg10, Atg12 and Atg16) and Atg8 (Atg3, Atg4, Atg7 and Atg8) conjugation systems involved in vesicle expansion.

In the budding yeast *Saccharomyces cerevisiae*, autophagy has been extensively studied and occurs either non-selectively or selectively with at least 40 autophagy-related genes (ATGs) [4]. Proteins related to autophagy from the plant and the human fungal pathogens and other industrially useful fungi are summarized in Table 1. Briefly, in selective autophagy process the autophagosome formation occurs via the assembly of a pre-autophagosomal structure (PAS), where organelles such as mitochondria (mitophagy), nuclei (nucleophagy), and peroxisomes (pexophagy) are encapsulated by a double membrane envelope derived from the endoplasmic reticulum (ER) (Figure 1). Subsequently, the autophagosomes fuse with the vacuoles, and autophagic degradation is mediated by vacuolar hydrolytic enzymes [5]. After the membranes of autophagic bodies are broken down by hydrolytic enzymes, degraded products are channeled back to the cytoplasm for reuse in metabolic and biosynthetic pathways [6]. ATGs that mediate non-selective and selective autophagy are classified into various groups based on their functions in the formation of phagophores and their maturation into autophagosomes [7]. This group includes the ATG1-ATG13-ATG17 complex which comprises Atg1, Atg11, Atg13, Atg17, Atg29, and Atg31, whose function is to scaffold essential sites required for autophagosome initiation at the phagophore. The second group is vital for phagophore expansion and is a membrane delivery system including Atg2, Atg9, and Atg18. The third group is mainly for vesicle nucleation and is part of the phosphatidylinositol 3-kinase complex (Vps34, Vps15, Vps30/Atg6, and Atg14). Finally, the vesicle expansion group is a ubiquitin-like conjugation system including Atg5, Atg7, Atg10, Atg12, and Atg16. In this review we provide an overview of the multiple roles of autophagy (macroautophagy) in various fungi including the plant, human, and industrial fungal species and summarize the functions of characterized ATGs in various fungal species.

## 2. Selective Autophagy Mechanisms in *Saccharomyces cerevisiae*

In *S. cerevisiae* the cytoplasm-to-vacuole (Cvt) pathway transports hydrolases, specifically aminopeptidase I (Ape1), from the cytoplasm to the vacuole as part of the selective autophagy mechanism. The Ape1 complex binds to the Atg19 receptor to form the Cvt complex, and the Atg19 receptor interacts with the adaptor protein Atg11. Atg11 accumulates on PAS by interacting with Atg1 and Atg9. During mitophagy induction, the transmembrane mitophagy receptor Atg32, localized on the mitochondrial outer membrane [60,61,62], interacts with Atg11. Atg11 tethers the mitochondria to the PAS for the selective sequestration of the mitochondria by the isolation membrane (Figure 1A). The endoplasmic reticulum–mitochondria encounter structure (ERMES) complex acts as a physical tether between the ER and mitochondria and aids in expanding the isolation membrane to provide lipid sources from ER and extension of the isolation membrane, leading to mitophagosome formation and its eventual fusion with the vacuole for degradation by vacuolar hydrolases.

While the factors participating in the autophagic degradation of nuclear components in other organisms remain largely unknown, *S. cerevisiae* has a unique nucleophagy transmembrane receptor protein, Atg39, localized on the nuclear envelope [63] (Figure 1B). Atg39 interacts with Atg8 through an Atg8-interacting motif in its N-terminal cytosolic tail, and upon nucleophagy induction, it binds to Atg8 puncta in an Atg8-dependent manner to form the nucleophagosome [64]. While Atg39 is essential for the selective autophagy of proteins within the outer and inner nuclear membrane, nucleolus, and nucleoplasm [63,64], it is not required for the autophagic degradation of nuclear pore components [65,66,67]. Instead, recent studies have identified nucleoporin Nup159 as an Atg8-binding protein that promotes the autophagic degradation of nuclear pore components [65,66].

The crucial importance of pexophagy in degrading impaired peroxisomes is exemplified by the fact that peroxisomes maintain cellular homeostasis in response to oxidative stress and play major role in lipid metabolism, including fatty acid β-oxidation [68,69]. In *S. cerevisiae*, the peroxisomal membrane protein Pex3 acts as a peroxisomal ligand and is central to initiating pexophagy by facilitating the recruitment of the Atg36 receptor protein to the peroxisomal membrane (Figure 1C). Atg36 binds to the adaptor protein Atg11 and recruits peroxisomal fission complexes containing the dynamin-related GTPases Dnm1 and Vps1 to target peroxisomes, thereby facilitating their sequestration by phagophores [70].

In addition to the above established canonical selective autophagic pathways, a Golgi membrane-dependent intracellular proteolytic process called the Golgi membrane-associated degradation (GOMED) pathway has also been reported [71]. Previously, GOMED was classified as alternative autophagy due to its morphological and functional similarities with canonical autophagy. However, recent studies have shown differences between the two processes in terms of the protein machinery involved and the degraded substrates and their biological roles [72].

## 3. Autophagy Genes Regulate Multiple Processes in Plant and Human Pathogenic Fungi, and Industrial Fungi

### 3.1. ATG1-ATG13-ATG17 Complex of Autophagy Genes

ATG1-ATG13-ATG17 complex autophagy genes include *Atg1*, *Atg11*, *Atg13*, *Atg17*, and *Atg29* and are known to regulate the induction of autophagosome formation. Most of these genes have been characterized in fungi and have been shown to control various fungal developmental processes. In the plant fungal pathogens *Magnaporthe oryzae*, *Ustilago maydis*, *Botrytis ceinerea*, and *Sclerotina sclerotiorum*, *Atg1* was reported to be crucial for autophagy and essential for fungal developmental processes including conidiation, conidial germination, turgor generation, and fungal pathogenesis [8,10,11,26,73]. However, in *Fusarium graminearum*, although there was severe defect in autophagy, the Δ*FgAtg1* mutant strain still caused infection in wheat [9]. In the human fungal pathogen *Aspergillus fumigatus*, the Δ*AfAtg1* deletion strain was attenuated in sporulation but retained virulence in a murine aspergillosis infection model [19]. However, in *C. neoformans* Ding et al. reported *Atg1* as being indispensable for virulence in a murine cryptococcosis model [17]. The exact role of *Candida albicans Atg1* in conidiation and virulence is unclear, although its importance has been demonstrated for biofilm formation [74]. *Atg11*, *Atg13*, *Atg17*, and *Atg29* genes also have been shown to perform various functions in fungal developmental processes. In *M. oryzae*, the *MoAtg13* mutant strain had no effects on appressorial penetration; however, it exhibited reduced virulence. While the Δ*MoAtg11* and Δ*MoAtg29* mutant strains sporulated and remained pathogenic like the wild-type strain, Δ*MoAtg17* had penetration defects, but it still remained nonpathogenic [26]. While in *S. sclerotiorum* loss of *SsAtg17* impaired sclerotia development and fungal virulence [28], in *F. graminearum* the Δ*FgAtg17* deletion strain remained virulent on the host plant [9]. In *U. Maydis*, *UmAtg11* was reported to be involved in mitophagy, but similar to the Δ*MoAtg11* mutant, the Δ*UmAtg11* mutant strain remained unaffected in its ability to cause infection in the host plant [21]. However, in the human fungal pathogens *C. albicans* and *C. neoformans*, *Atg11* deletion strains displayed contrasting function as the deletion of *Atg11* in *C. albicans* remarkably reduced conidiation and pathogenesis [25] and completely abolished pathogenesis in *C. neoformans* [24]. These studies indicate various roles for the Atg1/ULK complex autophagy genes in fungal developmental and pathogenesis.

**Figure 2:**
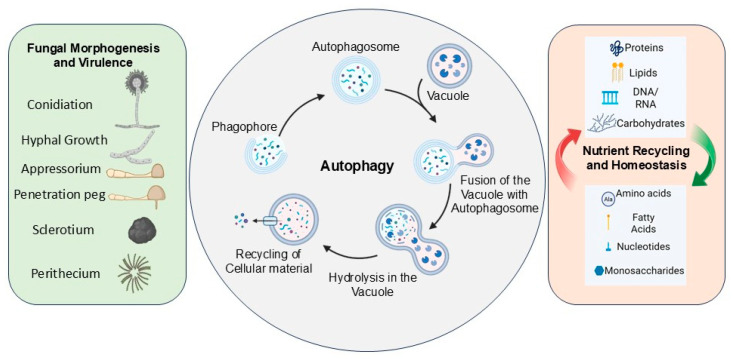
**Schematic diagram of fungal autophagy process and its roles in morphogenetic events and nutrient homeostasis**. **Center panel**—Following the induction of autophagy, from the pre-autophagosomal structure a cup-shaped double-membrane phagophore is formed that eventually seals to form a mature autophagosome, with the cellular cargo material destined for degradation, and matures into an autophagosome. Following fusion of the autophagosome with the vacuole, the vacuolar enzymes hydrolyze the cellular cargo, and the degraded products are recycled in the cell. **Right side panel**—The conversion of complex cellular proteins, lipids, nucleic acids, and carbohydrates into simpler compounds including amino acids, fatty acids, nucleotides, and monosaccharides, respectively. **Left side panel**—The various morphogenetic events, including growth and differentiation processes of conidiation, appressoria, sclerotia and perithecia formation, that are impacted by autophagy.

Figure 2 illustrates general autophagy process in funsgi and its multifunctional roles in fungal morphogenetic events, and nutrient recycling and homeostasis.

### 3.2. The Phosphatidylinositol 3-Kinase (PtdIns3K) Complex of Autophagy Genes

The (PI3K) complex comprises genes such as *Atg6*, *Atg14*, *Vps15*, and *Vps34* that are important for the induction of autophagosome formation, extension, and expansion of phagosomes. Also in this category are the *Vps38* and *Vps15* genes that are involved in the endocytic pathway. In *M. oryzae*, deletion of the (PI3K) complex genes *Atg6* and *Atg14* resulted in conidiation defects, and the Δ*MoAtg6* and Δ*MoAtg14* mutant strains appressoria lost the ability to penetrate the host plant and eventually led to loss of virulence [26]. Similarly, Liu et al. showed that *Botrytis cinerea BcAtg6* is important in the regulation of autophagy, and its deletion contributed to defects in mycelial growth, conidiation, and sclerotia formation [29]. Furthermore, silencing of *Phytophthora sojae PsAtg6a* significantly reduced sporulation and pathogenicity with the *PsAtg6a*-silenced strain displaying haustoria formation defects [30]. In other fungi such as *F. graminearum*, *Ustilaginoidea virens* and *C. neoformans*, Atg14 was reported to impact fungal conidiation and pathogenesis [9,24,32]. These findings suggest that *Atg6* and *Atg14* have conserved roles in controlling fungal developmental processes.

### 3.3. ATG9 Trafficking Autophagy Genes

The ATG9 trafficking autophagy genes are important for phagophore expansion, and the *Atg2*, *Atg9*, and *Atg18* genes have also been shown to be critical for fungal developmental processes. In *M. oryzae*, deletion of *MoAtg2*, *MoAtg9*, and *MoAtg18* impaired fungal conidiation and pathogenesis [26]. Also, in other fungal pathogens such as *S. sclerotiorum*, *F. graminearum*, and *B. cinerea*, loss of function of *Atg2* led to defects in conidiation and pathogenesis as their deletion mutants exhibited reduced sporulation and virulence [9,16,33]. Although the function of *Colletotrichum fructicola* Atg9 in growth and conidiation remains unknown, loss of function of the *CfAtg9* gene caused defects in mitosis and attenuated appressoria formation, appressoria turgor pressure, and virulence, indicating its importance for autophagy and pathogenicity [35]. Just as in plant pathogenic fungi, the *Atg9* genes have been reported in the human fungal pathogen *C. neoformans* to control conidiation and pathogenesis [36].

### 3.4. Ubiquitin-like System of Autophagy Genes

The ubiquitin-like system of autophagy-related genes play crucial roles in the extension and expansion of autophagosomes and include *Atg3*, *Atg5*, *Atg7*, *Atg8*, *Atg10*, *Atg12*, and *Atg16* genes. Majority of the ubiquitin-like system genes have been reported to be involved in non-selective autophagy in the plant fungal pathogen *M. oryzae* [26]. Knockout mutants of *MoAtg3*, *MoAtg5*, *MoAtg7*, *MoAtg8*, *MoAtg10*, *MoAtg12*, *MoAtg16* displayed defects in host penetration and lost virulence [26,75]. In a genome-wide association study, 28 ATGs were identified in *F. graminearum* [9]. Deletion of ubiquitin-like system genes *FgAtg3*, *FgAtg5*, *FgAtg7*, *FgAtg8*, *FgAtg10*, *FgAtg12*, and *FgAtg16* significantly reduced sporulation and virulence [9]. *B. cinerea*, *BcAtg3* and *BcAtg7,* and *BcAtg8* were also reported to be crucial for the autophagy process as both single and deletion mutant strains; Δ*BcAtg3*, Δ*BcAtg7*, and Δ*BcAtg8* were defective in mycelial growth, conidiation, sclerotia formation, and pathogenesis [38,76]. In other plant fungal pathogens *Aspergillus flavus*, *S. sclerotiorum*, * Colletotrichum* species, and the industrial fungus *Aspergillus oryzae*, Atg8, which is an important marker of autophagy, is pivotal for various processes including conidiation, appressoria formation, appressoria turgor pressure, and virulence [41,47,48,49,77]. The role of the ubiquitin-like system of autophagy-related genes has also been elucidated in human fungal pathogens. Zhao et al. generated 22 ATG deletion strains in *C. neoformans* and established that the mutant strains including Δ*Atg5*, Δ*Atg7*, Δ*Atg8*, Δ*Atg12* and Δ*Atg16*, were attenuated in virulence [24]. In addition, *C. neoformans Atg8* RNAi knockdown mutant strain was also reported to be reduced in virulence in a murine model [51]. Based on these studies, it is evident that the ubiquitin-like system of autophagy genes plays almost similar roles in controlling growth, conidiation, and virulence in different fungal species.

## 4. Multifunctional Roles for Autophagy in Fungi

### 4.1. Autophagy in Nutrient Recycling, Homeostasis, Cellular Differentiation, and Degradation

As detailed in Section 3, the various groups of autophagy genes involved in the formation of the autophagosome, membrane delivery, and vesicle nucleation and expansion regulate multiple functions in diverse fungi. In addition to key processes involving growth and differentiation, autophagy genes have also been shown to be essential for pathogenic traits involving host penetration and infection. During these processes, the stress induced by host factors may be a contributing factor to the induction of autophagy.

In addition to controlling the morphogenetic and developmental aspects in various fungal species, autophagy also plays a critical role in nutrient recycling pathways in several fungal species. For instance, in *M. oryzae* when the Δ*MoAtg1* mutant with blocked autophagy was cultured on nutrient-deficient minimal media, including nitrogen and carbon, the mutant was attenuated in growth when compared to the wild-type strain [8]. Also, the Δ*MoAtg8* mutant of *M. oryzae* was significantly reduced in sporulation, which was restored upon the addition of alternative carbon sources, glucose or sucrose, or glucose-6-phosphate [78,79]. The *Aspergillus fumigatus* Δ*AfAtg1* mutant that was deficient in autophagy exhibited limited growth on nutrient starvation medium (i.e., water-agarose) when compared with the wild-type strain and the complemented strains that exhibited normal growth on the starvation medium [19]. However, when the mutant strain was transferred back to rich medium, growth was restored, indicating that the growth defect was due to lack of nutrients.

In addition to nutrient recycling, metal ion homeostasis impacts autophagy. For instance, depletion of cations such as zinc, manganese, and iron induces autophagy [19]. These studies offer clear evidence on the role of autophagy-dependent nutrient recycling and metal ion homeostasis in fungal growth and development. In eukaryotes, autophagy serves as the key process for protein degradation, where long-lived proteins and whole damaged or obsolete organelles are degraded [80]. Unlike animals, filamentous fungi lack lysosomes; therefore, vacuoles play similar role in the degradation process. In *A. oryzae*, hyphal vacuolation was reported to rapidly increase in the mycelia of *A. oryzae* under nutrient-starved conditions [81]. In *A. fumigatus* and *M. oryzae*, autophagic bodies were present in the vacuoles of their respective wild-type strains upon autophagy induction but absent in Δ*AfAtg1* and Δ*MoAtg1* [8,19]. Lastly, during deletion of *MoYpt7*, the Rab GTPase, and *MoMon1*, the guanine nucleotide exchange factor for Ypt7 blocked autophagy and altered vacuole assembly and vacuole fusion in the Δ*MoMon1* and Δ*MoYpt7* deletion strains [82,83]. These studies provide supportive evidence on the role of autophagy in cellular degradation.

Autophagy plays a key role in cellular differentiation, and mutants blocked in autophagy display various defects in growth and development. In *M. oryzae*, the autophagy-deficient Δ*MoAtg8* mutant strain was attenuated in sporulation and conidiation, being significantly suppressed upon supplementation of exogenous glucose or sucrose; however, appressoria lost the ability to penetrate the plant tissue [78]. Deletion of *MoAtg1*, *MoAtg4*, and *MoAtg5* resulted in reduced conidiation, impaired germination, reduced appressorium turgor pressure, with the appressoria of Δ*MoAtg1*, Δ*MoAtg4*, and Δ*MoAtg5* mutant strains losing their ability to penetrate the host plant tissue [8,75,84]. Loss of *Atg8* function in *C. orbiculare* led to defects in germination and appressoria development [46]. In *B. cinerea*, deletion of *BcAtg1* led to defects in growth, conidiation, sclerotial development, appressoria formation, and penetration [11]. Perithecia formation during sexual reproduction is affected by deletion of the autophagy-related vacuolar protease (PspA) gene in the plant fungal pathogen *Podospora anserina* [85]. Autophagy has been shown to cause and prevent cell death in different fungi. For instance, in the plant pathogenic fungus *M. oryzae*, deletion of *MoAtg14* blocked autophagy, and staining of the Δ*MoAtg14* mutant strain spores with fluorescein diacetate to verify spore viability showed that a high percentage of Δ*MoAtg14* mutant cells were still alive compared to the wild-type strain, indicating that *MoAtg14* contributes to conidial cell death [31]. In addition, the DNA-binding E3 ubiquitin-protein ligase *Snt2* deletion in *M. oryzae* affected autophagy homeostasis and fungal cell death, with Δ*MoSnt1* compromising development of infection structure, conidiation, oxidative stress tolerance, and cell wall integrity. [86]. Also, in *Magnaporthe grisea*, autophagy was shown to be indispensable for spore collapse (cell death) during *in planta* infection [79]. However, in *P. anserina* autophagy is dispensable for cell death, though it tends to be stimulated during cell death by incompatibility [87]. Moreover, autophagy is linked to autolysis, which is a natural self-degradation process orchestrated by endogenous hydrolases, and prolonged autolysis has been shown to result in cell death [88].

### 4.2. Autophagy in Antifungal Drug Resistance and Response Mechanisms

Antifungal drugs specifically target the key components of the fungal cells, the cell wall, and the cell membrane, compromise their integrity, and induce cellular damage [89]. Autophagy can be activated as a protective response to drug stress, degrading damaged cellular components, and mitigating oxidative stress, thereby promoting drug tolerance [90,91]. Due to the established roles for autophagy in fungal stress adaptation, it has recently gained significance in antifungal drug resistance processes. Antifungal drug resistance is a complex process that evolves through a multitude of factors, resulting in fungal adaptation to antifungal drug exposure [92]. Enhanced activity of drug efflux systems, including membrane transport proteins, overexpression of drug resistance-related genes involved in ergosterol and glucan synthesis, the key components of fungal cell membranes and cell walls, respectively, and biofilm formation to counteract the stress imposed by antifungals, are the major components operating to induce resistance. For instance, previous studies in the autophagy-deficient strains of *C. albicans* have shown reduced expression of *Cdr1* and *Mdr1* efflux pump genes, indicating the impact of autophagic processes on drug efflux activity [93].

Antifungals induce cellular stress that results in autophagic response. In this regard, the azoles (fluconazole, itraconazole), the polyene antibiotic amphotericin B, and the DNA synthesis inhibitor nystatin have been shown to induce the formation of autophagosomes in *C. albicans* biofilms [94]. Interestingly, Huang et al. recently showed that a combination of antifungals (amphotericin B or 5-fluorocytosine) with aspirin suppressed biofilm formation by activating autophagy and inhibiting TOR signaling [74]. Strategies to target autophagy-related processes/proteins that are fungal-specific are necessary to overcome the challenges of increasing drug resistance observed with current clinical antifungals, including the azoles and echinocandins. Previous studies have shown promise in this direction of designing specific inhibitors to autophagy proteins. During autophagy induced by starvation or rapamycin, a selective inhibitor, autophinib, has been established to target Vps34 [95]. Another autophagy modulator, berberine, has been shown to synergize with fluconazole treatment and cause growth inhibition of an azole-resistant strain of *C. albicans* [96].

## 5. Regulation of Autophagy

### 5.1. Kinase-Phosphatase Modules Regulating Autophagy Machinery

Thus far, three kinases including TOR kinase, Atg1, and protein kinase A (PKA) have been identified to phosphorylate a set of autophagy-related proteins in *S. cerevisiae*. TOR is a multifunctional Ser/Thr protein kinase regulating cellular growth and development, nutrient acquisition, and protein synthesis and is important for nutrient signaling and autophagy. TORC1 phosphorylates *S. cerevisiae* Atg1, Atg13 and Atg29 proteins. During normal growth conditions, active TOR phosphorylates Atg13, which in turn modulates Atg1 activity. However, under nitrogen starvation conditions, TOR is inactivated, resulting in reduced phosphorylation of Atg13, leading to an increased affinity of Atg13 for Atg1 and stimulating the formation of Atg1–Atg13 complex required for autophagy induction [97,98]. Atg1 is also a Ser/Thr protein kinase and functions downstream of TOR, regulating different steps in autophagosome formation. Atg1 is autophosphorylated [99] and has been shown to phosphorylate other autophagy proteins including Atg4, Atg9, Atg13 and Atg29. For instance, Atg1-dependent phosphorylation of Vps34 is required for robust autophagy activity in *S. cerevisiae*, and Vps34-dependent atg1 phosphorylation was shown to be important for full autophagy activation and cell survival [100]. Similarly, Atg9 was reported to be phosphorylated by Atg1, and this phosphorylation of Atg9 is crucial for regulation of early stages in autophagy through efficient recruitment of Atg8 and Atg18 to the site of autophagosome formation and subsequent expansion of the isolation membrane [101]. Although Atg1-mediated phosphorylation of Vps34 and Atg9 promotes the autophagy process, Sanchez et al. showed that phosphorylation of Atg4 by Atg1 blocks autophagy orchestrated by Atg4 [102]. These results demonstrate that Atg1-mediated phosphorylation of autophagy-related genes regulates autophagy in a positive and negative manner.

In contrast to *S. cerevisiae*, not much is known about the kinases regulating the phosphorylation of ATG proteins in filamentous fungi. We previously reported PKA-dependent phosphorylation of several autophagy-related proteins, including ATG proteins Atg20 and Atg24, and the vacuolar sorting proteins, including Vps1 (VpsA), Vps15, Vps17, Vac8, and Vtc4 [58]. Vps1 is a dynamin-like GTPase that plays a critical role in the transport of Atg9-containing vesicles to the pre-autophagosomal structure, and its role in pexophagy has also been reported [103]. Vps15 is a regulatory protein of Vps34 and is referred to as a pseudokinase as it does not contain the characteristic catalytic domain residues present in protein kinases. Although Vps15 does not phosphorylate Vps34, it forms a complex with it and is required for Vps34 activity in autophagy. Vac8 is a vacuolar armadillo repeat protein shown to be required for efficient induction of nonselective autophagy in *S. cerevisiae*. Localization of Vac8 to the vacuole is required for Atg1 initiation complex recruitment and pre-autophagosomal structure assembly at the vacuoles. Vac8 acts as an anchor at the vacuole and binds to Atg13, which forms an assembly hub for the recruitment of the initiation complex. Interestingly, the deletion of *Vac8* in *S. cerevisiae* did completely abolish autophagy but significantly impacted autophagic activity [104]. Vtc4 belongs to the vacuolar transport chaperone complex of proteins that have recently been found to negatively regulate endocytosis and autophagy [105]. In *M. oryzae*, *MoYck1*, which encodes casein kinase, a Ser/Thr protein kinase, was shown to negatively regulate the autophagy process. Loss of *Moyck1* function affected growth, conidiation, conidial germination, and appressorium formation and penetration. Examination of GFP-MoAtg8 revealed a faster degradation in the Δ*Moyck1* background compared with the wild-type strain. Furthermore, GFP-*MoATG8* showed elevated levels in the Δ*Moyck1* background, indicating MoYck1 negatively controls autophagy [106]. Type PP2C phosphatases, Ptc2 and Ptc3, have also been shown to regulate autophagy in the yeast through interaction and dephosphorylation of the Atg1 complex. Loss of function of these phosphatases inhibits starvation-induced macroautophagy and the cytoplasm-to-vacuole targeting pathway via impairing the assembly of the essential autophagy machinery to the phagophore [107]. Similarly, Kondo et al. showed that Cdc14 protein phosphatase plays an important role in the induction of autophagy following starvation and the target of rapamycin complex 1 (TORC1) kinase inactivation through rapid dephosphorylation of Atg13 [108]. Also, Cdc14 was necessary for effective induction of ATG8 and ATG13 expression [108]. These studies clearly demonstrate that autophagy is regulated through phosphorylation and dephosphorylation in fungal species.

### 5.2. Other Effectors Involved in the Regulation of Autophagy

Besides phosphorylation, autophagy is also regulated through acetylation. The Sin3 histone deacetylase complex (Sin3-HDAC) was shown as a transcriptional repressor of ATGs. Sin3 thus negatively regulates autophagy induction in *M. oryzae* [109]. Loss of function of Sin3 resulted in upregulations of ATGs and promoted autophagy. Moreover, Wu et al. established that Sin3 negatively regulated the transcription of Atg1, Atg13, and Atg17 through direct occupancy and histone acetylation. During nutrient starvation conditions, the transcription of Sin3 was downregulated, and the reduced occupancy of Sin3 from those ATGs resulted in hyperacetylation and activated their transcription which subsequently promoted autophagy.

In addition, dynamins, which are a large superfamily of GTPase proteins and function as motor proteins, have also shown to be important for autophagy. In *M. oryzae*, MoDnm1 was found to localize in the peroxisomes and mitochondria and is essential for vegetative growth, conidiogenesis, and full pathogenicity [110]. MoDnm1 was also shown to interact with the mitochondrial fission protein MoFis1 and the WD adaptor protein MoMdv1. The importance of the MoFis1, MoMdv1 and MoDnm1 complexes in autophagy was analyzed by monitoring Pex14 (pexophagy marker), Porin (mitophagy marker), and Atg8 (autophagy marker). Fewer stable Pex14 and porin proteins were observed in the Δ*MoDnm1*, Δ*MoFis1*, and Δ*MoMdv1* mutant strains relative to the wild-type strain. Furthermore, the expression of Atg8-GFP to visualize autophagic bodies in the background of these three mutations revealed lesser accumulation of autophagic bodies in the lumen of their vacuoles in comparison to the wild-type, confirming their roles in autophagy.

## 6. Summary and Future Prospects

Research on autophagy in fungi has been extensively undertaken in the past two decades following the identification of ATGs in yeast [111]. Majority of these studies have clearly elucidated the involvement of autophagy processes in fungal development and pathogenicity. In this review, we broadly reviewed the roles of various ATGs in a wide variety of fungal species, including plant and human pathogenic fungi and industrially useful fungi. More importantly, majority of the ATGs have a conserved role in different fungal species and can be exploited for antifungal design. Although most of the studies have only evaluated their involvement in fungal development and pathogenesis, observations on how autophagy regulates fungal development and pathogenesis and the molecular mechanism involved remain unclear and call for further investigation, if autophagy has to be exploited for therapeutic purposes.

## Figures and Tables

**Table 1:** List of autophagy-related genes characterized in plant, human and industrial fungi.

Gene Name	Organism	Conidiation	Pathogenesis	Reference
	ATG1-ATG13-ATG17 Complex			
*MoAtg1*	*Magnaporthe oryzae*	Reduced	Reduced	[8]
*FgAtg2*	*Fusarium graminearum*	Reduced	Reduced	[9]
*UmAtg1*	*Ustilago maydis*	No effect	Reduced	[10]
*BcAtg1*	*Botrytis cinerea*	Reduced	Reduced	[11,12]
*AnAtg1*	*Aspergillus niger*	Reduced	NA	[13]
*BbAtg1*	*Beauveria bassiana*	Reduced	Reduced	[14]
*BdAtg1*	*Botryosphaeria dothidea*	Reduced	Reduced	[15]
*SsAtg1*	*Sclerotinia sclerotiorum*	Reduced	Reduced	[16]
*CnAtg1*	*Cryptococcus neoformans*	Unknown	Reduced	[17]
*CaAtg1*	*Candida albicans*	Unknown	Unknown	[18]
*AfAtg1*	*Aspergillus fumigatus*	Reduced	Normal	[19]
*MoAtg11*	*Magnaporthe oryzae*	No effect	No effect	[20]
*UmAtg11*	*Ustilago maydis*	Unknown	No effect	[21,22]
*AoAtg11*	*Aspergillus oryzae*	Unknown	NA	[23]
*CnAtg11*	*Cryptococcus neoformans*	Unknown	Abolished	[24]
*CaAtg11*	*Candida albicans*	Reduced	Reduced	[25]
*MoAtg13*	*Magnaporthe oryzae*	Reduced	Abolished	[26]
*AoAtg13*	*Aspergillus oryzae*	Reduced	NA	[23]
*CaAtg13*	*Candida albicans*	Unknown	Unknown	[27]
*MoAtg17*	*Magnaporthe oryzae*	No effect	No effect	[26]
*FgAtg17*	*Fusarium graminearum*	No effect	Reduced	[9]
*SsAtg17*	*Sclerotinia sclerotiorum*	Unknown	Reduced	[28]
*AnAtg17*	*Aspergillus niger*	No effect	NA	[13]
	**PtdIns3K Complex**			
*FgAtg6*	*Fusarium graminearum*	Reduced	Reduced	[9]
*BcAtg6*	*Botrytis cinerea*	Reduced	Reduced	[29]
*PsAtg6a*	*Phytophthora sojae*	Reduced	Reduced	[30]
*CnAtg6*	*Cryptococcus neoformans*	Unknown	Abolished	[24]
*MoAtg14*	*Magnaporthe oryzae*	Reduced	Abolished	[31]
*FgAtg14*	*Fusarium graminearum*	Reduced	Reduced	[9]
*UvAtg14*	*Ustilaginoidea virens*	Reduced	Reduced	[32]
*CnAtg14-03*	*Cryptococcus neoformans*	Unknown	Abolished	[24]
	**ATG9 Trafficking Complex**			
*MoAtg2*	*Magnaporthe oryzae*	Reduced	Abolished	[26]
*FgAtg2*	*Fusarium graminearum*	Reduced	Reduced	[9]
*BcAtg2*	*Botrytis cinerea*	Reduced	Abolished	[33]
*PlAtg2*	*Peronophythora litchii*	Reduced	Reduced	[34]
*SsAtg2*	*Sclerotinia sclerotiorum*	Unknown	Reduced	[16]
*MoAtg9*	*Magnaporthe oryzae*	Reduced	Abolished	[20]
*CfAtg9*	*Colletotrichum fructicola*	Unknown	Reduced	[35]
*FgAtg9*	*Fusarium graminearum*	Reduced	Reduced	[9]
*CaAtg9*	*Candida albicans*	Unknown	Normal	[36]
*FgAtg18*	*Fusarium graminearum*	Reduced	Reduced	[9]
*FgAtg27*	*Fusarium graminearum*	No effect	No effect	[37]
	**Ubiquitin-like system Complex**			
*MoAtg3*	*Magnaporthe oryzae*	Reduced	Abolished	[26]
*FgAtg3*	*Fusarium graminearum*	Reduced	Reduced	[9]
*BcAtg3*	*Botrytis cinerea*	Reduced	Reduced	[38]
*BdAtg3*	*Botryosphaeria dothidea*	Reduced	Reduced	[39]
*FoAtg3*	*Fusarium oxysporum*	Reduced	Reduced	[40]
*MoAtg4*	*Magnaporthe oryzae*	Reduced	Abolished	[26]
*CfAtg4*	*Colletotrichum fructicola*	Reduced	Reduced	[41]
*FgAtg4*	*Fusarium graminearum*	Reduced	Reduced	[9]
*AoAtg4*	*Aspergillus oryzae*	Abolished	NA	[23]
*CpAtg4*	*Cryphonectria parasitica*	Reduced	Reduced	[42]
*SsAtg4*	*Sclerotinia sclerotiorum*	Unknown	Reduced	[16]
*CnAtg4*	*Cryptococcus neoformans*	Unknown	Unknown	[43]
*MoAtg5*	*Magnaporthe oryzae*	Reduced	Abolished	[26]
*FgAtg5*	*Fusarium graminearum*	Reduced	No effect	[9]
*SsAtg5*	*Sclerotinia sclerotiorum*	Unknown	Reduced	[16,28]
*CnAtg5*	*Cryptococcus neoformans*	Unknown	Reduced	[24]
*MoAtg7*	*Magnaporthe oryzae*	Reduced	Abolished	[26]
*FgAtg7*	*Fusarium graminearum*	Reduced	Reduced	[9]
*BcAtg7*	*Botrytis cinerea*	Reduced	Reduced	[38]
*UvAtg7*	*Ustilaginoidea virens*	Reduced	Reduced	[44]
*CnAtg7*	*Cryptococcus neoformans*	Unknown	Reduced	[17,45]
*MoAtg8*	*Magnaporthe oryzae*	Reduced	Abolished	[26]
*CoAtg8*	*Colletotrichum orbiculare*	Unknown	Abolished	[46]
*CfAtg8*	*Colletotrichum fructicola*	Unknown	Abolished	[35]
*CsAtg8*	*Colletotrichum scovillei*	Reduced	Reduced	[47]
*FgAtg8*	*Fusarium graminearum*	Reduced	Reduced	[9]
*UmAtg8*	*Ustilago maydis*	No effect	Reduced	[10]
*BcAtg8*	*Botrytis cinerea*	Reduced	Reduced	[38]
*AfAtg8*	*Aspergillus flavus*	Reduced	Reduced mycotoxin	[48]
*AoAtg8*	*Aspergillus oryzae*	Abolished	NA	[49]
*UmAtg8*	*Ustilago maydis*	No effect	Reduced	[10]
*BcAtg8*	*Botrytis cinerea*	Reduced	Reduced	[38]
*AfAtg8*	*Aspergillus flavus*	Reduced	Reduced mycotoxin	[48]
*AnAtg8*	*Aspergillus niger*	Reduced	NA	[13]
*FoAtg8*	*Fusarium oxysporum*	Reduced	Reduced	[50]
*AnAtg8*	*Aspergillus niger*	Reduced	NA	[13]
*FoAtg8*	*Fusarium oxysporum*	Reduced	Reduced	[50]
*CnAtg8*	*Cryptococcus neoformans*	Unknown	Reduced	[51]
*MoAtg10*	*Magnaporthe oryzae*	Reduced	Abolished	[26]
*FgAtg10*	*Fusarium graminearum*	Reduced	Reduced	[9]
*FgAtg12*	*Fusarium graminearum*	Reduced	Reduced	[9]
*SmAtg12*	*Sordaria macrospora*	Reduced	Unknown	[52]
*SsAtg12*	*Sclerotinia sclerotiorum*	Unknown	Reduced	[28]
*CnAtg12*	*Cryptococcus neoformans*	Unknown	Reduced	[24]
*CgAtg16*	*Colletotrichum gloeosporioides*	Reduced	Reduced	[53]
*FgAtg16*	*Fusarium graminearum*	Reduced	Reduced	[9]
*CnAtg16*	*Cryptococcus neoformans*	Unknown	Reduced	[24]
	**Other groups**			
*CoAtg26*	*Colletotrichum orbiculare*	Not explained	Abolished	[46]
*ChAtg24*	*Colletotrichum higginsianum*	Reduced	Reduced	[54]
*FgAtg15*	*Fusarium graminearum*	Reduced	Reduced	[55]
*AoAtg15*	*Aspergillus oryzae*	No effect	NA	[56]
*AoAtg26*	*Aspergillus oryzae*	Reduced	NA	[57]
*CnAtg15*	*Cryptococcus neoformans*	Unknown	Reduced	[24]
*CnAtg20*	*Cryptococcus neoformans*	Unknown	Reduced	[24]
*CnAtg24*	*Cryptococcus neoformans*	Unknown	Reduced	[24]
*CnAtg15*	*Cryptococcus neoformans*	Unknown	Reduced	[24]
*CaAtg27*	*Candida albicans*	Unknown	Unknown	[27]
*AfAtg20*	*Aspergillus fumigatus*	Reduced	Reduced	[58]
*AfAtg24*	*Aspergillus fumigatus*	Reduced	Reduced	[59]

**Footnote:** NA is Not Applicable.

## Data Availability

No new data were created or analyzed in this study. Data sharing is not applicable.
